# Layperson-Delivered Telephone-Based Behavioral Activation Among Low-Income Older Adults During the COVID-19 Pandemic: The HEAL-HOA Randomized Clinical Trial

**DOI:** 10.1001/jamanetworkopen.2024.16767

**Published:** 2024-06-18

**Authors:** Jojo Yan Yan Kwok, Da Jiang, Dannii Yuen-lan Yeung, Namkee G. Choi, Rainbow Tin Hung Ho, Lisa Marie Warner, Kee-Lee Chou

**Affiliations:** 1 School of Nursing, The University of Hong Kong, Pokfulam, Hong Kong; 2 Centre on Behavioral Health, The University of Hong Kong, Pokfulam, Hong Kong; 3 Department of Special Education and Counselling, The Education University of Hong Kong, Tai Po, Hong Kong; 4 Department of Social and Behavioural Sciences, City University of Hong Kong, Kowloon Tong, Hong Kong; 5 Steve Hicks School of Social Work, University of Texas at Austin, Austin, Texas; 6 Department of Social Work & Social Administration, The University of Hong Kong, Pokfulam, Hong Kong; 7 Department of Psychology, MSB Medical School Berlin, Berlin, Germany; 8 Department of Social Sciences and Policy Studies, The Education University of Hong Kong, Tai Po, Hong Kong

## Abstract

**Importance:**

Older adults are particularly vulnerable to loneliness and its physical and psychosocial sequelae, but scalable interventions are lacking, especially during disasters such as pandemics.

**Objective:**

To compare the effects of layperson-delivered, telephone-based behavioral activation and mindfulness interventions vs telephone-based befriending on loneliness among at-risk older adults.

**Design, Setting, and Participants:**

This assessor-blinded, 3-arm randomized clinical trial screened Chinese older adults through household visits and community referrals from April 1, 2021, to April 30, 2023, in Hong Kong. Eligible participants (≥65 years of age) who were lonely, digitally excluded, living alone, and living below the poverty line and provided consent to participate were randomized into behavioral activation, mindfulness, and befriending groups. Assessments were conducted at baseline, 1 month, and 3 months.

**Intervention:**

As part of the Helping Alleviate Loneliness in Hong Kong Older Adults (HEAL-HOA) dual randomized clinical trial, 148 older laypersons were trained to deliver a twice-weekly 30-minute intervention via telephone for 4 weeks.

**Main Outcomes and Measures:**

The primary outcome was loneliness measured by the UCLA Loneliness Scale (range, 20-80) and the De Jong Gierveld Loneliness Scale (range, 0-6), with higher scores on both scales indicating greater loneliness. Secondary outcomes were depression, perceived stress, life satisfaction, psychological well-being, sleep quality, perceived social support, and social network.

**Results:**

A total of 1151 participants (mean [SD] age, 76.6 [7.8] years; 843 [73.2%] female) were randomized to the behavioral activation (n = 335), mindfulness (n = 460) or befriending (n = 356) group. Most were widowed or divorced (932 [81.0%]), had primary education or below (782 [67.9%]), and had 3 or more chronic diseases (505 [43.9%]). Following intention-to-treat principles, linear mixed-effects regression model analyses showed that loneliness measured by the UCLA Loneliness Scale was significantly reduced in the behavioral activation group (mean difference [MD], −1.96 [95% CI, −3.16 to −0.77] points; *P* < .001]) and in the mindfulness group (MD, −1.49 [95% CI, −2.60 to −0.37] points; *P* = .004) at 3 months compared with befriending. Loneliness measured by the De Jong Gierveld Loneliness Scale was not significantly reduced at 3 months in the behavioral activation group (MD, −0.06 [95% CI, −0.26 to 0.13] points; *P* > .99]) but was in the mindfulness group (MD, 0.22 [95% CI, 0.03 to 0.40] points; *P* = .01) at 3 months compared with befriending. In the behavioral activation and mindfulness groups, sleep quality improved compared with befriending, but perceived stress increased. Psychological well-being and perceived social support improved in the behavioral activation group. No statistically significant between-group differences were observed in depression, life satisfaction, or social network.

**Conclusion and Relevance:**

In this randomized clinical trial, scalable psychosocial interventions delivered remotely by older laypersons appeared promising in reducing later life loneliness and addressing the pressing mental health challenges faced by aging populations and professional geriatric mental health workforce shortages. Further research should explore ways to maximize the clinical relevance and cost-effectiveness of these interventions.

**Trial Registration:**

Chinese Clinical Trial Registry Identifier: ChiCTR2300072909

## Key Points

**Question:**

Were telephone-based behavioral activation and mindfulness interventions delivered by older laypersons superior to telephone-based befriending in reducing loneliness among low-income older adults during the COVID-19 pandemic?

**Findings:**

In this randomized clinical trial of 1151 older adults who were lonely, digitally excluded, living alone, and living below the poverty line, 4-week layperson-delivered, telephone-based interventions significantly reduced UCLA Loneliness Scale and De Jong Gierveld Loneliness Scale scores for the behavioral activation and mindfulness groups compared with the telephone-based befriending group at 3 months after the intervention.

**Meaning:**

These results suggest that older layperson–delivered, telephone-based structured psychosocial interventions, particularly behavioral activation and mindfulness, hold promise as effective, safe, and accessible options for addressing loneliness in at-risk older adults.

## Teaser

This randomized clinical trial compares the effects of layperson-delivered, telephone-based behavioral activation and mindfulness interventions with telephone-based befriending on loneliness during the COVID-19 pandemic among at-risk older adults.

## Introduction

Loneliness is a significant global health concern, particularly among older adults who face reduced mobility and limited social contacts.^1^ The COVID-19 pandemic and related public health measures, such as the physical distancing ordinance and suspension of community-based psychosocial services, further intensified isolation and its impact on health and well-being,^2,3^ making it crucial to develop scalable, accessible, and effective interventions. Psychosocial interventions targeting maladaptive social cognition and connectedness, including behavioral activation and mindfulness, have shown promise among community-dwelling older adults but required well-trained counselors.^4^ In view of the strain on the health and social care workforce during the COVID-19 pandemic, layperson-delivered, telephone-based psychosocial interventions have emerged as a potential solution.^5,6,7,8^ However, empirical evidence for these interventions targeting late-life loneliness during the pandemic is scant. To our knowledge, no randomized clinical trial (RCT) has tested the pragmatic effectiveness of remote psychosocial interventions for older adults delivered by peer counselors who were older adults themselves. In response to the existing knowledge gap, shortage of health and social care workforce, and specific challenges posed by COVID-19, we developed and implemented a territory-wide initiative known as the “Helping Alleviate Loneliness in Hong Kong Older Adults” (HEAL-HOA) dual RCT (investigating both volunteer intervention deliverers and participants of these interventions), which aimed to alleviate loneliness among at-risk older adults. In this HEAL-HOA project, we rapidly trained older laypersons (ages 50-70 years) to provide remote, telephone-based psychosocial interventions to their peers facing heightened loneliness risks, particularly those who had reported elevated loneliness, living alone and in poverty, and lacking digital access.

This study is the first, to our knowledge, to compare the effects of layperson-delivered, telephone-based behavioral activation and mindfulness interventions vs telephone-based befriending on loneliness (primary outcome) and associated mental health outcomes among socioeconomically disadvantaged and lonely older adults during the COVID-19 pandemic. Befriending was chosen as the treatment-as-usual control condition as it is a common social care practice in the Hong Kong community, and it provides support for participants to develop an emotion-focused relationship but does not teach any skills on behavioral activation or mindfulness. We hypothesized that participants in telephone-based behavioral activation and/or mindfulness would experience greater reductions in loneliness, stress, and symptoms of depression and anxiety and improvements in life satisfaction, psychological well-being, sleep quality, social support, and social network compared with participants in telephone-based befriending, both immediately after the intervention (time 1, 4 weeks after baseline) and at 3-month follow-up (time 2, 2 months after completing the 4-week intervention).

## Methods

### Study Design

As part of the HEAL-HOA dual RCT, this study was a pragmatic, assessor-blinded, 3-arm RCT comparing telephone-based behavioral activation and mindfulness to telephone-based befriending among older adults who experienced loneliness, were socioeconomically disadvantaged, lived alone, and lacked access to the internet at home. The trial protocol is published^9^ and is given in Supplement 1. The trial was approved by the Human Research Ethics Board of The Education University of Hong Kong. All participants provided written informed consent, and all data were anonymous. The study was prospectively registered in the Clinical Trials Registry of The University of Hong Kong Clinical Trials Centre (HKUCTR-2929) as well as retrospectively registered at the Chinese Clinical Trial Registry in the WHO Registry Network (ChiCTR2300072909); identical content is in both registries. This report follows the 2010 Consolidated Standards of Reporting Trials (CONSORT) reporting guideline and its extension to nonpharmacological interventions.^10^

### Participants

To ensure a diverse sample, we recruited participants from public housing estates primarily catering to solo-living older adults, community centers for older adults, and older adult academies via convenience sampling between April 1, 2021, and April 30, 2023, in Hong Kong. We also established collaborations with community centers for older adults and older adult academies and distributed the study flyers or directly approached potential participants during group meetings in these centers. Additionally, we used email invitations for older adult participants in the service units and classrooms of older adult academies.

The study inclusion criteria were age 65 years or older, feelings of loneliness (ie, ≥6 on the 3-item UCLA Loneliness Scale),^11^ monthly household income less than US $577 (ie, half of the median household income for single-person households), and proficiency of Cantonese via telephone. The exclusion criteria were cognitive impairment, psychiatric disorders, access to the internet at home, and engagement in regular mindfulness or related mind-body practices at baseline (>2 times in each week). As a token of appreciation, participants received a financial incentive in the form of a US $31 supermarket voucher on completion of the intervention and all assessments.

### Screening, Baseline Testing, Randomization, and Blinding

All assessors, interventionists, and the older adult study participants were blinded to the experimental vs control condition and to the study hypotheses. The informed consent form provided participants with general information about investigating loneliness and psychosocial well-being, without disclosing specific intervention details or claiming superiority of any intervention. The number of intervention groups was also not disclosed.

After undergoing baseline assessment, eligible participants were randomly assigned to the behavioral activation, mindfulness, or befriending groups, using a 1:1:1 allocation ratio. The randomization sequence was generated by an independent research assistant using an online random number generator. The details of group allocation were concealed using sequentially numbered identifiers. While blinding was not possible for participants and interventionists due to the nature of the interventions, research assistants conducting baseline and follow-up assessments remained blinded to the group assignments.

### Fidelity Monitoring

To ensure accuracy and adherence, a fidelity monitoring process was implemented. This process involved accurately recording the duration and number of sessions for each call, documenting any specific details or difficulties encountered during the intervention, and closely examining cases in which participants discontinued their involvement. During the fidelity monitoring process, a substantial number of participants (n = 130) assigned to the mindfulness group dropped out between January 1 and April 30, 2022. This dropout was due to the implementation of a stringent physical distancing ordinance in Hong Kong during that period.^12^ The face-to-face mindfulness volunteer training coincided with this period, resulting in an extended waiting period for participants in the telephone-based mindfulness group to receive the intervention. To achieve the planned sample size per group (ie, at least 322 per group), the research team adjusted the allocation ratio of behavioral activation, mindfulness, and befriending to 1:2:1 starting April 1, 2022, resulting in a final imbalanced sample (n = 335:460:356).

### Interventions

As part of the HEAL-HOA dual RCT, a total of 375 volunteers 50 to 70 years of age who felt lonely were recruited to test the effects of volunteering. These participants were assigned to either the volunteer group (n = 185), where they delivered the intervention to the study participants, or the active control condition (n = 190), which involved a psycho-education program along with social gatherings. Baseline characteristics, including age, sex, and loneliness, did not exhibit significant differences among the 3 groups of volunteers. The effects of volunteering on the volunteers themselves have been reported elsewhere.^13^ A total of 148 volunteers were randomized and trained to deliver telephone-based behavioral activation (n = 45), mindfulness (n = 48), or befriending (n = 55) interventions. The training involved six 2-hour face-to-face weekly sessions conducted in small groups (4-6 participants) led by an experienced social worker and a research assistant. The training sessions included lectures, practice sessions, and role plays that were developed by the research team. Volunteers received a stipend of US $25 on completing the training program. Each trained volunteer interventionist consistently delivered 8 twice-weekly 30-minute sessions of the assigned intervention for each study participant over a period of 6 months, with the number of participants per volunteer ranging from 1 to 10.

#### Telephone-Based Behavioral Activation

Telephone-based behavioral activation was adapted from the Telehealth Behavioral Activation Treatment Manual for Homebound Older Adults with Depression.^14^ It aimed to help participants understand the link between behavior and mood, increase positive and healthy behaviors, and decrease negative and unhealthy behaviors to improve mood and manage loneliness and social isolation. Specific behavioral activation steps included the identification of life areas and values that they want to focus on, selection of activity goals in the chosen life areas (eg, increasing social connectedness), and planning activities to accomplish the goals by identifying and addressing potential barriers to implementing the selected activities. Volunteers also played an active role in changing participants’ negative cognitive processes leading to inactivity. Details of the intervention are given in eTable 1 in Supplement 2.

#### Telephone-Based Mindfulness

Telephone-based mindfulness was adapted from a smartphone-delivered mindfulness intervention developed by Lindsay et al.^15^ to alleviate loneliness in stressed adults. This intervention aimed to reduce loneliness by teaching participants 2 mindfulness skills: present-focused awareness and monitoring and acceptance. The intervention covered the key concepts of mindfulness, including skills for body scanning, focusing on the body and mind, cultivating equanimity, recognizing bodily sensations, as well as guidance on maintaining a positive attitude toward stress. Details of the intervention are given in eTable 2 in Supplement 2.

#### Telephone-Based Befriending

Telephone-based befriending served as a treatment as usual control condition and was adapted from BEFRIENDAS, the Befrienders Information Guide developed by the National Aging Research Institute.^16^ Unlike behavioral activation and mindfulness, befriending focused on providing emotional support rather than teaching specific psychosocial skills. In befriending, volunteers called participants twice a week. Each telephone call was planned for 30 minutes, but participants had the freedom to end the call at any point. Volunteers introduced themselves and initiated casual conversations covering a wide range of topics, such as weather, television programs, current news, and memorable recent events. Participants had the choice to continue or switch to another topic.

### Outcome Measures

All outcome measures were assessed at baseline (time 0), 4 weeks (time 1, immediately after the intervention), and 3 months (time 2, which was 2 months after the intervention). Baseline assessment was conducted in person, while time 1 and time 2 follow-up assessments were conducted over the telephone. At baseline, we asked participants to report demographic characteristics (age, sex, marital status, and level of education) and the number of chronic diseases, as these variables were found to be associated with loneliness in older adults.^17,18,19^

The primary outcome was loneliness measured with the revised UCLA Loneliness Scale (UCLA-L; Chinese version)^20^ and the De Jong Gierveld Loneliness (DJGL) Scale (Chinese).^21^ These 2 scales are widely used for measuring loneliness in older adults.^22^ The UCLA-L measures the frequency of 20 symptoms on a scale from 0 (no symptoms) to 3 (always). The DJGL measures the agreement with 6 items related to participants’ current situations and feelings, with neutral and positive answers scored as 1 for negatively worded questions. The total score ranges for UCLA-L and DJGL scales are 20 to 80 and 0 to 6, respectively, with higher scores implying greater loneliness. To our knowledge, no minimal clinically important differences have been established for either scale.

Secondary outcomes included (1) depressive symptoms measured by the Patient Health Questionnaire-9 (Chinese),^23^ with scores ranging from 0 to 27 and higher scores indicating greater depressive symptom severity; (2) anxiety measured by the Hospital Anxiety and Depression Scale-Anxiety Subscale,^24^ with scores ranging from 0 to 21 and higher scores indicating greater symptom severity in the past week; (3) psychological distress measured by the 14-item Perceived Stress Scale (Chinese),^25^ which assesses stress level by evaluating the degree to which an individual has perceived life as unpredictable, uncontrollable, and overloading in the past month, with scores ranging from 0 to 56, with higher scores indicating higher levels of stress; (4) life satisfaction measured by the Satisfaction With Life Scale (Chinese),^26^ in which participants were asked to rate their agreement with statements related to subjective well-being and life satisfaction on a scale from 1 to 7; scores range from ranges from 5 to 35, with higher scores indicating higher levels of life satisfaction; (5) psychological well-being measured by the Psychological Well-Being Scale (Chinese),^27^ which measures domains related to autonomy, environmental mastery, personal growth, positive relations with others, purpose in life, and self-acceptance; score range from 16 to 96, with higher scores indicating higher levels of psychological well-being; (6) sleep quality measured by the Sleep Condition Indicator (Chinese version)^28^; scores range from 0 to 32, with higher scores indicating better sleeping quality; (7) perceived social support measured by the Multidimensional Scale of Perceived Social Support^25^; scores range from from 12 to 84, with higher scores indicating a higher perception of social support from family, friends, and significant others, and (8) social network measured by the Lubben Social Network Scale^29^; scores range from 0 to 30, with higher scores indicating more social engagement.

### Sample Size

Our sample size calculation was based on comparing participants allocated to behavioral activation and those allocated to befriending. We chose this comparison because, based on previous videoconference and laypersondelivered behavioral activation,^5,6^ we anticipated that behavioral activation would be more effective than befriending. In a previous behavioral activation trial with the UCLA-L Scale as the primary outcome measure,^6^ behavioral activation compared with friendly visit showed an effect size (Cohen *d*) of 0.5, favoring behavioral activation. To be conservative, we assumed that behavioral activation in this study would produce an effect size of 0.25, and we needed 289 participants in each group for a 2-sided test with an α of .05 and β of .10. Considering potential attrition in telephone-based interventions during the pandemic, a minimum of 322 participants was needed in each group to allow for up to 10% loss to follow-up.

### Statistical Analysis

We used descriptive statistics to compare the baseline characteristics of participants in the 3 arms. The 2 main comparisons were behavioral activation vs befriending and mindfulness vs befriending. An intention-to-treat (ITT) analysis was performed for all randomized participants who had at least 1 outcome measurement taken. To assess the intervention effects on the primary and secondary outcomes, mixed-effects linear regression was used, with the intercept taken as random, and time (as a continuous variable), group (using befriending as the reference), and group by time interaction included as independent variables. Linear contrasts were used to obtain between-group differences. Multiplicity due to multiple comparisons among the 3 groups at the 2 follow-up time points was accounted for using the Bonferroni approach.^30^ Normality of the residuals and random effects was assessed using normal probability plots. Missing values were not replaced because mixed-effects models can accommodate participants with at least 1 outcome measurement.^31^ Per-protocol sensitivity analysis was conducted by including participants who completed at least 75% (ie, 6 of 8) of the assigned sessions and completed the follow-up assessments. To assess the potential influence of a missing-not-at-random mechanism, additional sensitivity analyses were conducted using pattern-mixture models.^32^ We used SPSS version 29.0 (SPSS Inc) and Stata version 14.1 (StataCorp LLC) software for statistical analyses. To account for multiplicity, a 1.25% (ie, *P* < .01 after rounding) level of significance was assumed, and all significance tests were 2-sided.

## Results

The Figure shows participant flow through the study. We screened 4152 older adults through household visits in 102 public housing estates (n = 3467) and referrals from 42 community centers (n = 519) and 4 academic institutions (n = 166) from April 1, 2021, through April 30, 2023. Among 1917 eligible older adults, 1151 consented to participate (consent rate, 60.0%) and were randomized into the behavioral activation (n = 335), mindfulness (n = 460), and befriending (n = 356) groups. Table 1 shows the baseline demographic characteristics of participants. The mean (SD) age of participants was 76.6 (7.8) years; 843 (73.2%) were female and 308 (26.8%) were male. The majority of participants were widowed or divorced (932 [81.0%]), had primary education or below (782 [67.9%]), and were living with 3 or more chronic diseases (505 [43.9%]). The groups were generally well balanced, although the mindfulness group comprised relatively more women.

**Figure.**
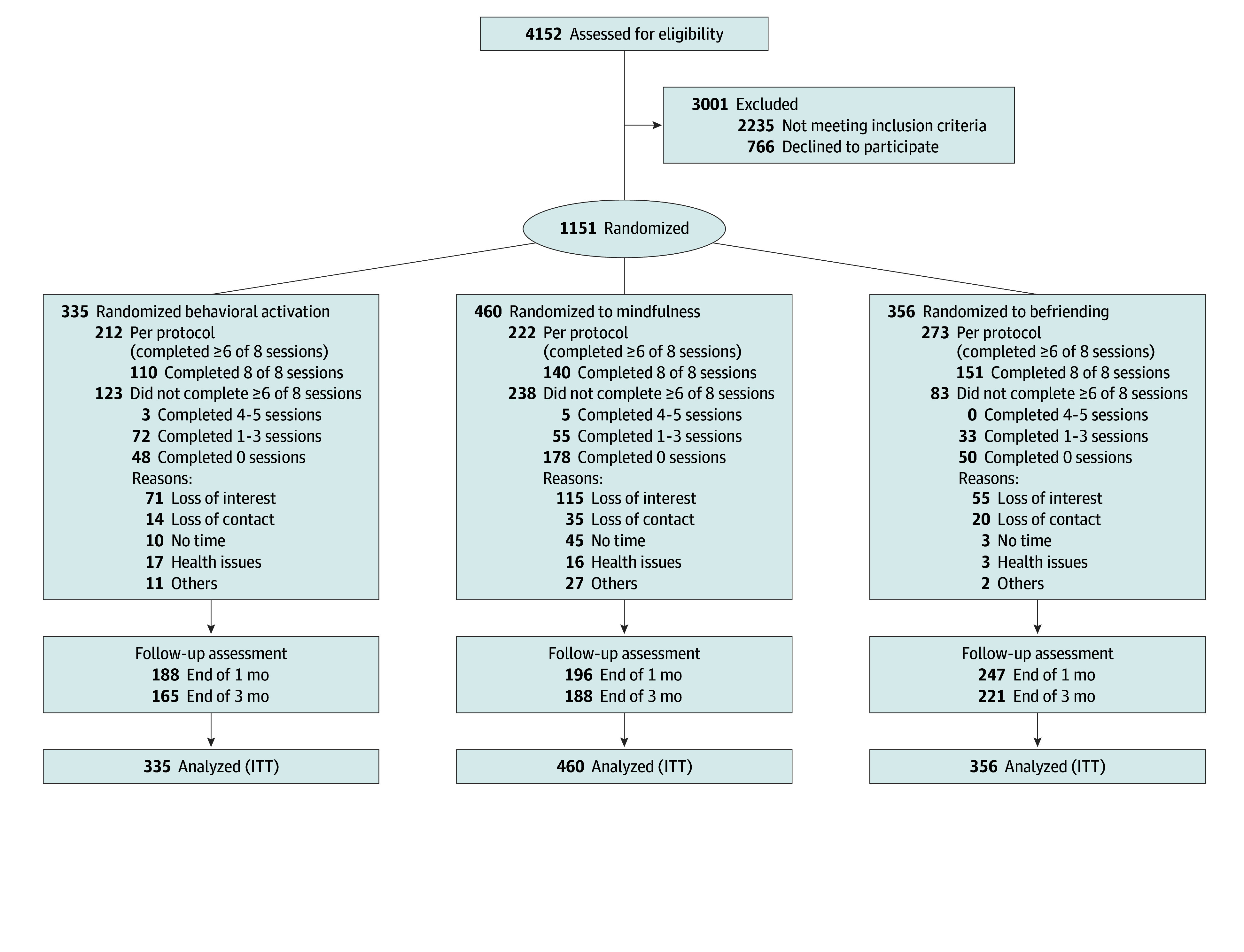
Patient Flow Diagram ITT indicates intention to treat.

**Table 1.** Baseline Demographic and Clinical Characteristics^a^

Characteristic	Participants, No (%)		
	Behavioral activation (n = 335)	Mindfulness (n = 460)	Befriending (n = 356)
Age, mean (SD), y	75.3 (7.0)	77.4 (8.1)	76.7 (8.1)
Sex			
Female	247 (73.7)	354 (77.0)	242 (68.0)
Male	88 (26.3)	106 (23.0)	114 (32.0)
Marital status			
Divorced	115 (34.3)	121 (26.3)	93 (26.1)
Married	17 (5.1)	33 (7.2)	25 (7.0)
Single (never married)	50 (14.9)	43 (9.3)	51 (14.3)
Widowed	153 (45.7)	263 (57.2)	187 (52.5)
Level of education			
None	75 (22.4)	169 (36.7)	90 (25.3)
≤Primary	118 (35.2)	184 (40.0)	146 (41.0)
Secondary	118 (35.2)	98 (21.3)	110 (30.9)
Tertiary	24 (7.2)	9 (2.0)	10 (2.8)
No. of chronic diseases^b^			
0	53 (15.8)	88 (19.1)	45 (12.6)
1	71 (21.2)	78 (17.0)	66 (18.5)
2	68 (20.3)	102 (22.2)	75 (21.1)
≥3	143 (42.7)	192 (41.7)	170 (47.8)
UCLA-L, mean (SD)	50.4 (7.3)	51.6 (6.6)	51.5 (7.9)
DJGL, mean (SD)	4.5 (1.4)	4.8 (1.4)	4.3 (1.4)
PHQ-9, mean (SD)	2.0 (3.5)	2.5 (3.9)	2.6 (4.0)
HADS-A, mean (SD)	1.7 (3.4)	2.5 (3.8)	2.3 (3.6)
PSS, mean (SD)	16.5 (11.7)	20.3 (10.8)	11.8 (11.1)
SWLS, mean (SD)	18.9 (4.1)	18.1 (4.0)	19.2 (3.5)
PWB, mean (SD)	56.9 (8.3)	55.1 (6.4)	55.9 (8.2)
SCI, mean (SD)	23.0 (7.7)	22.8 (7.7)	21.2 (8.5)
MSPSS, mean (SD)	41.0 (15.1)	37.9 (13.2)	38.1 (16.4)
LSNS-6, mean (SD)	8.2 (5.8)	7.2 (5.6)	8.5 (6.3)

Abbreviations: DJGL, De Jong Gierveld Loneliness Scale; HADS-A, Hospital Anxiety and Depression Scale-Anxiety Subscale; LSNS-6, Lubben Social Network Scale; MSPSS, Multidimensional Scale of Perceived Social Support; PHQ-9, 9-item Patient Health Questionnaire; PSS, Perceived Stress Scale; PWB, Psychological Well-being Scale; SCI, Sleep Condition Indicator; SWLS, Satisfaction With Life Scale; UCLA-L, UCLA Loneliness scale.

^a^ Scoring of measurement tools is described in the Methods section.

^b^ Included chronic nonspecific lung disease, cardiac disease, peripheral disease, stroke, diabetes, arthritis, and cancer.

Of 1151 participants, 631 (54.8%) completed the time 1 follow-up assessment, and 574 (49.9%) completed the time 2 follow-up assessment. The follow-up dropout rates were 43.9% (147 of 335 participants) for behavioral activation, 57.4% (264 of 460) for mindfulness, 30.6% (109 of 356) for befriending, and 45.2% (520 of 1151) overall at time 1, while dropout rates at time 2 had risen to 50.7% (170 of 335) for behavioral activation, 59.1% (272 of 460) for mindfulness, 37.9% (135 of 356) for befriending, and 50.1% (577 of 1151) overall at time 2. The Figure shows the attendance of each intervention. In the per-protocol analysis, there were 707 participants who completed 6 or more sessions of the assigned intervention. Of these participants, 573 were classified as having completed time 1 and time 2 assessments, and 134 were classified as having not completed either the time 1 or time 2 assessment. There were no significant differences in demographics and baseline characteristics between participants who completed vs did not complete the assessments (eTable 3 in Supplement 2), except that those who completed the assessments demonstrated a higher perceived stress level compared with those who did not.

### Primary Outcome

Table 2 shows the results of the ITT analyses. Statistically significant overall between-group differences were reported in loneliness as measured by both the UCLA-L and DJGL scales at both time points. Loneliness as measured by the UCLA-L Scale significantly decreased in the behavioral activation (group × time interaction, mean difference [MD], −1.96 [95% CI, −3.16 to −0.77] points; Cohen *d* = 0.45; *P* < .001) and mindfulness (group × time interaction, MD, −1.49 [95% CI, −2.60 to −0.37] points; Cohen *d* = 0.34; *P* = .004) groups compared with the befriending group (ie, treatment as usual) at time 2, yet no significant difference was noted at time 1 for either behavioral activation (group × time interaction, MD, −1.16 [95% CI, −0.26 to 0.13] points; Cohen *d* = 0.16; *P* < .06) or mindfulness (group × time interaction, MD, −0.96 [95% CI, −2.07 to 0.16] points, Cohen *d* = 0.28; *P* < .12).

**Table 2.** Mixed-Effects Analysis on Primary Outcome^a^

Measure	Estimates across all follow-up periods, mean (95% CI) points^b^^,^^c^						Behavioral activation vs befriending			Mindfulness vs befriending			Overall between-group difference, *P* value
	Behavioral activation (n = 335)	*P* value	Mindfulness (n = 460)	*P* value	Befriending (n = 356)	*P* value	MD (95% CI)^d^	Cohen *d*	MD *P* value	MD (95% CI)^e^	Cohen *d*	MD *P* value	
**UCLA-L^f^**													
Time 0	50.43 (49.66 to 51.21)	NA	51.63 (51.02 to 52.23)	NA	51.54 (50.72 to 52.36)		NA	NA	NA	NA	NA	NA	NA
Time 1	49.50 (48.79 to 50.20)	<.001	49.70 (49.09 to 50.30)	<.001	50.66 (49.97 to 51.34)	<.001	−1.16 (−2.36 to 0.05)	0.16	.06	−0.96 (−2.07 to 0.16)	0.28	.12	<.001
Time 2	48.92 (48.21 to 49.62)	<.001	49.40 (48.80 to 50.00)	<.001	50.88 (50.20 to 51.56)	.12	−1.96 (−3.16 to −0.77)	0.45	<.001	−1.49 (−2.60 to −0.37)	0.34	.004	<.001
**DJGL^g^**													
Time 0	4.50 (4.35 to 4.66)	NA	4.83 (4.71 to 4.96)		4.28 (4.13 to 4.42)		NA	NA	NA	NA	NA	NA	NA
Time 1	4.16 (4.04 to 4.28)	<.001	4.40 (4.30 to 4.51)	<.001	4.00 (3.89 to 4.12)	<.001	0.15 (−0.05 to 0.36)	0.06	.22	0.40 (0.21 to 0.59)	0.21	<.001	.005
Time 2	4.02 (3.91 to 4.14)	<.001	4.30 (4.20 to 4.40)	<.001	4.09 (3.97 to 4.20)	.22	−0.06 (−0.26 to 0.13)	0.39	>.99	0.22 (0.03 to 0.40)	0.12	.01	<.001

Abbreviations: DJGL, De Jong Gierveld Loneliness Scale; ITT, intention-to-treat analysis; MD, mean difference; NA, not applicable; UCLA-L, UCLA Loneliness Scale.

^a^ All participants analyzed according to allocation (n = 1151). Analyses followed intention-to-treat principles. All *P* values reported in the analysis are nonadjusted; *P* < .01 considered statistically significant.

^b^ Paired *t* test between scores at time 0 (baseline) and time 1 (1 month assessment) for each intervention group separately.

^c^ Paired *t* test between scores at time 0 (baseline) and time 2 (3 months assessment) for each intervention group separately.

^d^ Difference between behavioral activation and befriending.

^e^ Difference between mindfulness and befriending.

^f^ DJGL Scale ranges from 0 to 6, with higher scores indicating greater loneliness.

^g^ UCLA-L Scale ranges from 20 to 80, with higher scores indicating greater loneliness.

Loneliness as measured by the DJGL Scale significantly decreased in the befriending group compared with the mindfulness group at time 1 (group × time interaction for time 1, MD, 0.40 [95% CI, 0.21-0.59] points; Cohen *d* = 0.21; *P* < .001), and the difference diminished in size but remained statistically significant at time 2 (group × time interaction for time 2, MD, 0.22 [95% CI, 0.03-0.40] points; Cohen *d* = 0.12; *P* = .01). No significant differences in DJGL scores were reported between the behavioral activation and befriending groups at time 1 (group × time interaction for time 2, MD, 0.15 [95% CI, −0.05 to 0.36] points, Cohen *d* = 0.06; *P* = .22) or time 2 (group × time interaction for time 2, MD, −0.06 [95% CI, −0.26 to 0.13] points; Cohen *d* = 0.39; *P* > .99).

The results of per-protocol analyses (eTable 4 in Supplement 2) consistently showed significant findings for the UCLA-L Scale. However, no significant differences were found for the DJGL Scale when comparing the behavioral activation and mindfulness groups to the befriending group.

Table 2 and the eFigure in Supplement 2 show the within-group comparisons for 3 groups over time. All 3 groups showed statistically significant within-group reductions at time 1 in UCLA-L (eg, mean, 50.43 [95% CI, 49.66-51.21] points at time 0 vs mean, 49.50 [95% CI, 48.79-50.20] points; *P* < .001 at time 1 for behavioral activation) and DJGL scores (eg, mean, 4.50 [95% CI, 4.35-4.66] points at time 0 vs mean, 4.16 [95% CI, 4.04-4.28] points at time 1 for behavioral activation; *P* < .001). At time 2, only the behavioral activation and mindfulness groups demonstrated sustained reductions in DJGL (eg, mean 4.02 [95% CI, 3.91-4.14] points; *P* < .001 for behavioral activation) and UCLA-L (eg, mean, 48.92 [95% CI, 48.21-49.62] points; *P* < .001 for behavioral activation) scores.

### Secondary Outcomes

Table 3 and eTable 5 in Supplement 2 present the results of the ITT and per-protocol analyses. Statistically significant overall between-group differences were reported in all secondary outcomes except depressive symptoms. Both the behavioral activation (group × time interaction, MD, 2.30 [95% CI, 1.04-3.56] points, Cohen *d* = 0.39; *P* < .001) and mindfulness (mindfulness group × time interaction, MD, 2.22 [95% CI, 1.05-3.38] points, Cohen *d* = 0.40; *P* < .001) groups reported significant small- to medium-sized improvement in sleep quality compared with the befriending group at time 1, but the significant effect was not sustained through time 2. Participants in the behavioral activation group had a statistically significant medium-sized improvement in psychological well-being at time 2 (group × time interaction, MD, 2.17 [95% CI, 0.94-3.41] points, Cohen *d* = 0.48; *P* < .001) and a small-sized improvement in perceived social support at time 2 (group × time interaction, MD, 3.06 [95% CI, 1.04-5.08] points, Cohen *d* = 0.36; *P* = .001) compared with the befriending group. Participants in the mindfulness group reported statistically significant lower life satisfaction at time 1 compared with the befriending group (group × time interaction, MD, −0.58 [95% CI, −1.13 to −0.03] points; *P* = .03), yet the effect size was negligible (Cohen *d* = 0.02), suggesting minimal clinical significance of this finding. Surprisingly, the befriending group reported a significant greater reduction in perceived stress on the Perceived Stress Scale (ie, 6 scores were higher, indicating greater stress, in the intervention groups) at both time points compared with both the behavioral activation (group × time interaction at time 1, MD, 3.79 [95% CI, 1.96-5.62] points, Cohen *d* = 0.28; *P* < .001; and at time 2, MD, 3.30 [95% CI, 1.60-5.00] points, Cohen *d* = 0.25; *P* < .001) and mindfulness (group × time interaction at time 1, MD, 7.29 [95% CI, 5.59-8.99] points, Cohen *d* = 0.63; *P* < .001; and at time 2, MD, 6.51 [95% CI, 4.93-8.08] points, Cohen *d* = 0.54; *P* < .001) groups.

**Table 3.** Mixed-Effects Analysis on Secondary Outcomes (ITT)^a^

Measure	Estimates across all follow ups, mean (95% CI), points^b^^,^^c^						Behavioral activation vs befriending			Mindfulness vs befriending			Overall between-group difference, *P* value
	Behavioral activation (n = 335)	*P* value	Mindfulness (n = 460)	*P* value	Befriending (n = 356)	*P* value	MD (95% CI)^d^	Cohen *d*	MD *P* value	MD (95% CI)^e^	Cohen *d*	MD *P* value	
**PHQ-9** ^f^													
Time 0	2.01 (1.64 to 2.39)	NA	2.54 (2.18 to 2.90)	NA	2.60 (2.19 to 3.01)	NA	NA	NA	NA	NA	NA	NA	NA
Time 1	1.98 (1.63 to 2.33)	.63	2.33 (2.03 to 2.63)	<.001	2.61 (2.27 to 2.95)	.90	−0.63 (−1.23 to −0.04)	0.20	.03	−0.28 (−0.83 to 0.27)	0.15	.66	.06
Time 2	2.11 (1.82 to 2.41)	.03	2.45 (2.20 to 2.71)	.34	2.71 (2.42 to 3)	.10	−0.59 (−1.11 to −0.09)	0.19	.01	−0.26 (−0.73 to 0.21)	0.08	.55	.57
**HADS-A**^f^													
Time 0	1.75 (1.39 to 2.11)	NA	2.53 (2.19 to 2.88)	NA	2.26 (1.89 to 2.63)	NA	NA	NA	NA	NA	NA	NA	NA
Time 1	1.72 (1.42 to 2.02)	.71	2.29 (2.04 to 2.54)	<.001	2.29 (2.00 to 2.57)	.76	−0.56 (−1.07 to −0.06)	0.22	.02	0.00 (−0.47 to 0.48)	0.09	>.99	.03
Time 2	1.77 (1.51 to 2.03)	.44	2.32 (2.09 to 2.54)	.24	2.31 (2.05 to 2.56)	.62	−0.53 (−0.98 to −0.09)	0.20	.01	0.01 (−0.40 to 0.42)	0.01	>.99	.29
**PSS** ^f^													
Time 0	16.52 (15.27 to 17.77)	NA	20.26 (19.27 to 21.25)	NA	11.76 (10.60 to 12.91)	NA	NA	NA	NA	NA	NA	NA	NA
Time 1	14.99 (13.92 to 16.07)	.001	18.50 (17.58 to 19.41)	<.001	11.21 (10.16 to 12.25)	<.001	3.79 (1.96 to 5.62)	0.28	<.001	7.29 (5.59 to 8.99)	0.63	<.001	<.001
Time 2	14.20 (13.2 to 15.19)	<.001	17.40 (16.55 to 18.25)	<.001	10.89 (9.92 to 11.86)	<.001	3.30 (1.60 to 5.00)	0.25	<.001	6.51 (4.93 to 8.08)	0.54	<.001	<.001
**SWLS** ^f^													
Time 0	18.94 (18.50 to 19.38)	NA	18.14 (17.77 to 18.51)	NA	19.23 (18.86 to 19.60)	NA	NA	NA	NA	NA	NA	NA	NA
Time 1	19.63 (19.29 to 19.98)	<.001	19.07 (18.77 to 19.37)	<.001	19.65 (19.31 to 19.99)	<.001	−0.02 (−0.61 to 0.57)	0.08	>.99	−0.58 (−1.13 to −0.03)	0.02	.03	<.001
Time 2	20.10 (19.76 to 20.43)	<.001	19.58 (19.29 to 19.86)	<.001	19.66 (19.33 to 19.98)	<.001	0.44 (−0.13 to 1.01)	0.39	.19	−0.08 (−0.61 to 0.45)	0.26	>.99	<.001
**PWB** ^f^													
Time 0	56.90 (56.01 to 57.78)	NA	55.13 (54.54 to 55.72)	NA	55.92 (55.01 to 56.78)	NA	NA	NA	NA	NA	NA	NA	NA
Time 1	58.70 (57.96 to 59.44)	<.001	57.05 (56.41 to 57.68)	<.001	57.41 (56.69 to 58.13)	<.001	1.29 (0.02 to 2.55)	0.20	.05	−0.36 (−1.54 to 0.81)	0.01	>.99	.12
Time 2	59.37 (58.64 to 60.09)	<.001	57.73 (57.11 to 58.35)	<.001	57.19 (56.49 to 57.9)	<.001	2.17 (0.94 to 3.41)	0.48	<.001	0.54 (−0.61 to 1.69)	0.30	.78	<.001
**SCI** ^f^													
Time 0	23.00 (22.18 to 23.82)	NA	22.84 (22.13 to 23.55)	NA	21.21 (20.32 to 22.09)	NA	NA	NA	NA	NA	NA	NA	NA
Time 1	23.23 (22.49 to 23.96)	.11	23.14 (22.51 to 23.77)	.009	20.93 (20.21 to 21.64)	.09	2.30 (1.04 to 3.56)	0.39	<.001	2.22 (1.05 to 3.38)	0.40	<.001	.006
Time 2	23.28 (22.61 to 23.95)	.22	23.18 (22.61 to 23.75)	.14	21.18 (20.53 to 21.83)	.17	2.10 (0.96 to 3.23)	0.26	<.001	2.00 (0.94 to 3.05)	0.24	<.001	.97
**MSPSS** ^f^													
Time 0	40.96 (39.35 to 42.58)	NA	37.88 (36.68 to 39.09)	NA	38.06 (36.35 to 39.76)	NA	NA	NA	NA	NA	NA	NA	NA
Time 1	41.97 (40.67 to 43.28)	.004	40.09 (38.98 to 41.21)	<.001	39.69 (38.42 to 40.96)	<.001	2.28 (0.06 to 4.51)	0.12	.04	0.40 (−1.66 to 2.47)	0.08	>.99	.03
Time 2	42.76 (41.57 to 43.94)	<.001	41.48 (40.47 to 42.49)	<.001	39.70 (38.55 to 40.85)	.007	3.06 (1.04 to 5.08)	0.36	.001	1.78 (−0.09 to 3.65)	0.37	.07	<.001
**LSNS-6** ^f^													
Time 0	8.15 (7.53 to 8.77)	NA	7.20 (6.69 to 7.71)	NA	8.46 (7.81 to 9.12)	NA	NA	NA	NA	NA	NA	NA	NA
Time 1	8.51 (8.01 to 9.01)	.01	7.85 (7.42 to 8.27)	<.001	8.71 (8.23 to 9.19)	.07	−0.20 (−1.04 to 0.65)	0.02	>.99	−0.87 (−1.65 to −0.08)	0.1	.03	.07
Time 2	8.89 (8.45 to 9.33)	<.001	8.50 (8.13 to 8.87)	<.001	8.64 (8.22 to 9.07)	.88	0.25 (−0.50 to 0.99)	0.25	>.99	−0.14 (−0.83 to 0.55)	0.29	>.99	<.001

Abbreviations: DJGL, De Jong Gierveld Loneliness Scale; HADS-A, Hospital Anxiety and Depression Scale-Anxiety Subscale; ITT, intention to treat; LSNS-6, Lubben Social Network Scale; MD, mean difference; MSPSS, Multidimensional Scale of Perceived Social Support; NA, not applicable; PHQ-9, 9-item Patient Health Questionnaire; PSS, Perceived Stress Scale; PWB, Psychological Well-Being Scale; SCI, Sleep Condition Indicator; SWLS, Satisfaction With Life Scale; UCLA-L, UCLA Loneliness scale.

^a^ All participants analyzed according to allocation (n = 1151). All *P* values reported in the analysis are nonadjusted; *P* < .01 considered statistically significant.

^b^ Paired *t* test between scores at time 0 (baseline) and time 1 (1 month assessment) for each intervention group separately.

^c^ Paired *t* test between scores at time 0 (baseline) and time 2 (3 months assessment) for each intervention group separately.

^d^ Difference between behavioral activation and befriending.

^e^ Difference between mindfulness and befriending.

^f^ See the Methods section for range and interpretation of scale.

All 3 groups showed statistically significant within-group reductions in perceived stress, and improvement in life satisfaction, psychological well-being, and social support at time 1 and time 2 (Table 3; eFigure in Supplement 2). Only the mindfulness group showed significant within-group reductions in depressive symptoms and anxiety and enhancement in sleep quality at time 1.

### Adverse Events

Two participants from the mindfulness group died during the study period. However, these deaths were determined to be unrelated to the trial intervention because participants had unrestricted access to their usual health care and were not subjected to any lifestyle restrictions. No other adverse events were reported during the trial.

## Discussion

To our knowledge, this is the largest randomized clinical trial to date assessing the effects of telephone-based psychosocial interventions for loneliness among at-risk older adults. Telephone-based behavioral activation, mindfulness, and befriending interventions delivered by older adult volunteers were all effective in reducing immediate loneliness and reducing stress, and enhancing life satisfaction, psychological well-being, and social support among Hong Kong Chinese older adults who were lonely, living in poverty, and digitally excluded during the COVID-19 pandemic. Particularly, compared with befriending, behavioral activation and mindfulness showed additional benefits on improving sleep quality at 1 month and reducing loneliness at 3 months.

Previous studies on remote psychosocial interventions for late-life loneliness typically involved trained volunteers in their 20s and 30s.^5,6,7^ Our study demonstrates that teaching affect and cognitive regulation skills to at-risk older adults can be extended through telephone delivery by rapidly trained older adult volunteers.^13^ Compared with befriending, participants from behavioral activation and mindfulness groups reported delayed, greater reductions in their loneliness assessed by the UCLA-L Scale after 3 months. These effects, with small to moderate sizes (Cohen *d*, 0.34-0.45), were robust, as evidenced by the consistent findings from both ITT and per-protocol analyses. Consistent with the results of a 2023 network meta-analysis,^4^ the present findings for psychosocial interventions behavioral activation and mindfulness demonstrated the greatest therapeutic benefits in combating loneliness and associated distress in later life. Befriending, although widely used as a low-cost care service to provide social support,^33^ has been shown to have relatively minimal and temporary therapeutic effects on psychological outcomes.^34^ Our study provides empirical evidence in favor of implementing more structured psychocognitive approaches to target maladaptive psychological processes contributing to chronic late-life loneliness.

Interestingly, befriending participants showed greater but small reductions in loneliness as assessed by the DJGL Scale compared with the mindfulness at 1 month and 3 months. However, these effects became insignificant in per-protocol analysis. The differences in conceptualization of loneliness and the interactive nature of the interventions may have contributed to these noncomplementary findings between UCLA-L and DJGL scales.^35^ The UCLA-L Scale emphasizes affective aspects in loneliness, which may be more sensitive to the psychocognitive regulating effects of mindfulness. On the other hand, the DJGJ scale takes a more behavioral approach, such as assessing reliance and trust in others. In addition, the active interaction in befriending vs the more script-based mindfulness intervention may have influenced the social synergetic effects. Enhancing the interactive components of the mindfulness intervention, such as incorporating dyad meditation exercises, could be considered in future research.

In contrast to previous literature,^3,6,7,36^ behavioral activation and mindfulness groups reported nonsignificant between-group differences in anxiety and depression compared with befriending. These nonsignificant findings may be attributed to the low baseline Patient Health Questionnaire-9 and Hospital Anxiety and Depression Scale-Anxiety Subscale scores of the present study participants, who reported minimal symptoms of depression and anxiety. It is noteworthy that befriending showed a significant and greater reduction in perceived stress at both time points compared with both behavioral activation and mindfulness. However, this finding should be interpreted with caution due to the significantly imbalanced baseline values of perceived stress across the 3 groups. While efforts were made to adjust the baseline values in the linear mixed-effects models, it is possible that the potential influence of regression to the mean was not fully accounted for when interpreting the findings.^37^ Future studies should further investigate the effects of the interventions and their mechanistic interactions on stress to provide more conclusive results.

### Strengths and Limitations

This study has strengths and limitations. A major strength is that it was implemented during the COVID-19 pandemic, allowing for interventions specifically tailored to address the challenges faced by older adults during this critical time. However, the pandemic also presented challenges for participant enrollment and adherence. First, the overall dropout rates (time 1, 45.2%; time 2, 50.1%) were slightly higher than in previous studies on remote interventions among individuals with chronic diseases or mental illnesses (ranging from 24.1% to 40.0%).^38,39^ However, our rates are tolerable considering the older adult population and pandemic context. Second, adherence was lower in the interventions compared with the active control, highlighting the need for strategies to enhance adherence, such as incorporating motivational strategies and simplifying programs. Qualitative research could provide insights into participant experiences and factors influencing motivation and acceptability. Future trials should consider the trade-offs between using formally trained experts (ie, behavioral therapists or meditation instructors) vs lay volunteers for intervention delivery in terms of scalability and cost-effectiveness. Third, the active control intervention may have underestimated the effects of the intervention, as it provided unique support from same-generation volunteers. Finally, the study did not assess the dyadic relationship or collect information on participants’ COVID-19 experiences, such as exposure history or loss of family members. Future investigations could consider including these aspects to better understand their effect on treatment outcomes and adherence.

## Conclusions

This randomized clinical trial found that telephone-based psychosocial interventions delivered by older adult volunteers were effective in reducing loneliness and perceived stress and in enhancing life satisfaction, psychological well-being, and social support among digitally excluded and lonely older adults (≥65 years of age) living alone and below the poverty line during the COVID-19 pandemic. Compared with befriending, behavioral activation and mindfulness showed additional benefits on improving sleep quality after 1 month and reducing loneliness after 3 months, with comparable benefits related to life satisfaction and psychological well-being. The accessibility, scalability, and low-cost nature of these interventions hold promise for significantly improving the psychological well-being of older adults, particularly those most vulnerable. Further research should explore strategies to maximize the clinical relevance of these psychosocial benefits.

## Notes

Supplement 1.
Trial Protocol

Supplement 2. **eTable 1.** Session Content of Tele-BA Intervention **eTable 2.** Session Content of Tele-MF Intervention **eTable 3.** Baseline Demographic and Clinical Characteristics Between Non-Completers and Completers (Per Protocol) **eTable 4.** Mixed-Effects Analysis on Primary Outcomes (Per Protocol) **eTable 5.** Mixed-Effects Analysis on Secondary Outcomes (Per Protocol) **eFigure.** Outcome Scores by Intervention Groups Across Assessment (Marginal Means)

Supplement 3.
Data Sharing Statement

## References

[1] Dahlberg L, McKee KJ, Frank A, Naseer M. A systematic review of longitudinal risk factors for loneliness in older adults. Aging Ment Health. 2022;26(2):225-249. doi:10.1080/13607863.2021.1876638 33563024

[2] Su Y, Rao W, Li M, Caron G, D’Arcy C, Meng X. Prevalence of loneliness and social isolation among older adults during the COVID-19 pandemic: a systematic review and meta-analysis. Int Psychogeriatr. 2023;35(5):229-241. doi:10.1017/S1041610222000199 35357280

[3] Kwok JYY, Choi EPH, Wong JYH, . A randomized clinical trial of mindfulness meditation versus exercise in Parkinson’s disease during social unrest. NPJ Parkinsons Dis. 2023;9(1):7. doi:10.1038/s41531-023-00452-w 36681670 PMC9862216

[4] Yu DS, Li PW, Lin RS, Kee F, Chiu A, Wu W. Effects of non-pharmacological interventions on loneliness among community-dwelling older adults: a systematic review, network meta-analysis, and meta-regression. Int J Nurs Stud. 2023;144:104524. doi:10.1016/j.ijnurstu.2023.104524 37295285

[5] Choi NG, Caamano J, Vences K, Marti CN, Kunik ME. Acceptability and effects of tele-delivered behavioral activation for depression in low-income homebound older adults: in their own words. Aging Ment Health. 2021;25(10):1803-1810. doi:10.1080/13607863.2020.1783516 32693614 PMC7855933

[6] Choi NG, Pepin R, Marti CN, Stevens CJ, Bruce ML. Improving social connectedness for homebound older adults: randomized controlled trial of tele-delivered behavioral activation versus tele-delivered friendly visits. Am J Geriatr Psychiatry. 2020;28(7):698-708. doi:10.1016/j.jagp.2020.02.008 PMC8767809 32238297

[7] Kahlon MK, Aksan N, Aubrey R, . Effect of layperson-delivered, empathy-focused program of telephone calls on loneliness, depression, and anxiety among adults during the COVID-19 pandemic: a randomized clinical trial. JAMA Psychiatry. 2021;78(6):616-622. doi:10.1001/jamapsychiatry.2021.0113 PMC7903319 33620417

[8] Torous J, Myrick K, Aguilera A. The need for a new generation of digital mental health tools to support more accessible, effective and equitable care. World Psychiatry. 2023;22(1):1-2. doi:10.1002/wps.21058 PMC9840484 36640397

[9] Warner LM, Jiang D, Yeung DY, . Study protocol of the ‘HEAL-HOA’ dual randomized controlled trial: testing the effects of volunteering on loneliness, social, and mental health in older adults. Contemp Clin Trials Commun. 2024;38:101275. doi:10.1016/j.conctc.2024.101275 PMC10904923 38435428

[10] Schulz KF, Altman DG, Moher D; CONSORT Group. CONSORT 2010 statement: updated guidelines for reporting parallel group randomised trials. BMJ. 2010;340:c332. doi:10.1136/bmj.c332 PMC2844940 20332509

[11] Lin CY, Tsai CS, Fan CW, . Psychometric evaluation of three versions of the UCLA Loneliness Scale (full, eight-item, and three-item versions) among sexual minority men in Taiwan. Int J Environ Res Public Health. 2022;19(13):8095. doi:10.3390/ijerph19138095 PMC9265606 35805754

[12] Government of the Hong Kong Special Administrative Region: Press Releases. Government suitably relaxes social distancing measures. Accessed October 20, 2023. https://www.info.gov.hk/gia/general/202204/14/P2022041400773.htm

[13] Warner LM, Yeung DY, Jiang D, . Effects of volunteering over six months on loneliness, social and mental health outcomes among older adults: the HEAL-HOA Dual Randomized Controlled Trial. Am J Geriatr Psychiatry. 2024;32(5):598-610. doi:10.1016/j.jagp.2023.12.022 38199937

[14] Choi NG, DiNitto DM, Marti CN, Choi BY. Telehealth use among older adults during COVID-19: associations with sociodemographic and health characteristics, technology device ownership, and technology learning. J Appl Gerontol. 2022;41(3):600-609. doi:10.1177/07334648211047347 PMC8847316 34608821

[15] Lindsay EK, Young S, Brown KW, Smyth JM, Creswell JD. Mindfulness training reduces loneliness and increases social contact in a randomized controlled trial. Proc Natl Acad Sci U S A. 2019;116(9):3488-3493. doi:10.1073/pnas.1813588116 PMC6397548 30808743

[16] Doyle C, Bhar S, Fearn M, . The impact of telephone-delivered cognitive behaviour therapy and befriending on mood disorders in people with chronic obstructive pulmonary disease: a randomized controlled trial. Br J Health Psychol. 2017;22(3):542-556. doi:10.1111/bjhp.12245 28544504

[17] Luanaigh CÓ, Lawlor BA. Loneliness and the health of older people. Int J Geriatr Psychiatry. 2008;23(12):1213-1221. doi:10.1002/gps.2054 18537197

[18] Cohen-Mansfield J, Hazan H, Lerman Y, Shalom V. Correlates and predictors of loneliness in older-adults: a review of quantitative results informed by qualitative insights. Int Psychogeriatr. 2016;28(4):557-576. doi:10.1017/S1041610215001532 26424033

[19] Mushtaq R, Shoib S, Shah T, Mushtaq S. Relationship between loneliness, psychiatric disorders and physical health? a review on the psychological aspects of loneliness. J Clin Diagn Res. 2014;8(9):WE01-WE04. doi:10.7860/JCDR/2014/10077.4828 PMC4225959 25386507

[20] Chou KL, Jun LW, Chi I. Assessing Chinese older adults’ suicidal ideation: Chinese version of the Geriatric Suicide Ideation Scale. Aging Ment Health. 2005;9(2):167-171. doi:10.1080/13607860412331336805 15804635

[21] Leung GT, de Jong Gierveld J, Lam LC. Validation of the Chinese translation of the 6-item De Jong Gierveld Loneliness Scale in elderly Chinese. Int Psychogeriatr. 2008;20(6):1262-1272. doi:10.1017/S1041610208007552 18590603

[22] Bugallo-Carrera C, Dosil-Díaz C, Anido-Rifón L, Pacheco-Lorenzo M, Fernández-Iglesias MJ, Gandoy-Crego M. A systematic review evaluating loneliness assessment instruments in older adults. Front Psychol. 2023;14:1101462. 10.3389/fpsyg.2023.1101462 PMC10166865 37179898

[23] Yu X, Tam WW, Wong PT, Lam TH, Stewart SM. The Patient Health Questionnaire-9 for measuring depressive symptoms among the general population in Hong Kong. Compr Psychiatry. 2012;53(1):95-102. doi:10.1016/j.comppsych.2010.11.002 21193179

[24] Leung CM, Ho S, Kan CS, Hung CH, Chen CN. Evaluation of the Chinese version of the Hospital Anxiety and Depression Scale: a cross-cultural perspective. Int J Psychosom. 1993;40(1-4):29-34. 8070982

[25] Chou KL. Assessing Chinese adolescents’ social support: the multidimensional scale of perceived social support. Pers Individ Dif. 2000;28(2):299-307. doi:10.1016/S0191-8869(99)00098-7

[26] Sachs J. Validation of the Satisfaction With Life Scale in a sample of Hong Kong University students. Psychologia. 2003;46(4):225-234. doi:10.2117/psysoc.2003.225

[27] Chan D, Chan L-k, Sun I. Developing a brief version of Ryff’s scale to assess the psychological well-being of adolescents in Hong Kong. Eur J Psychol Assess. 2017;35:1-9.

[28] Wong ML, Lau KNT, Espie CA, Luik AI, Kyle SD, Lau EYY. Psychometric properties of the Sleep Condition Indicator and Insomnia Severity Index in the evaluation of insomnia disorder. Sleep Med. 2017;33:76-81. doi:10.1016/j.sleep.2016.05.019 28449911

[29] Chan SCY, Wong CC, Huang QL, Fung CK. The psychometric properties of the Lubben Social Network Scale (LSNS-6) and its associations with well-being indicators in Hong Kong older adults. Australas J Ageing. 2023;42(4):683-689. doi:10.1111/ajag.13214 37259258

[30] Li G, Taljaard M, Van den Heuvel ER, . An introduction to multiplicity issues in clinical trials: the what, why, when and how. Int J Epidemiol. 2017;46(2):746-755. 10.1093/ije/dyw320 28025257

[31] Twisk J, de Boer M, de Vente W, Heymans M. Multiple imputation of missing values was not necessary before performing a longitudinal mixed-model analysis. J Clin Epidemiol. 2013;66(9):1022-1028. doi:10.1016/j.jclinepi.2013.03.017 23790725

[32] Son H, Friedmann E, Thomas SA. Application of pattern mixture models to address missing data in longitudinal data analysis using SPSS. Nurs Res. 2012;61(3):195-203. doi:10.1097/NNR.0b013e3182541d8c 22551994

[33] Preston C, Moore S. Ringing the changes: the role of telephone communication in a helpline and befriending service targeting loneliness in older people. Ageing Soc. 2019;39(7):1528-1551. doi:10.1017/S0144686X18000120

[34] Siette J, Cassidy M, Priebe S. Effectiveness of befriending interventions: a systematic review and meta-analysis. BMJ Open. 2017;7(4):e014304. doi:10.1136/bmjopen-2016-014304 PMC5594212 28446525

[35] Penning MJ, Liu G, Chou PHB. Measuring loneliness among middle-aged and older adults: the UCLA and de Jong Gierveld Loneliness Scales. Soc Indic Res. 2013;118:1147-1166.

[36] Orgeta V, Brede J, Livingston G. Behavioural activation for depression in older people: systematic review and meta-analysis. Br J Psychiatry. 2017;211(5):274-279. doi:10.1192/bjp.bp.117.205021 28982660

[37] Morton V, Torgerson DJ. Effect of regression to the mean on decision making in health care. BMJ. 2003;326(7398):1083-1084. doi:10.1136/bmj.326.7398.1083 PMC1125994 12750214

[38] Meyerowitz-Katz G, Ravi S, Arnolda L, Feng X, Maberly G, Astell-Burt T. Rates of attrition and dropout in app-based interventions for chronic disease: systematic review and meta-analysis. J Med Internet Res. 2020;22(9):e20283. doi:10.2196/20283 PMC7556375 32990635

[39] Linardon J, Fuller-Tyszkiewicz M. Attrition and adherence in smartphone-delivered interventions for mental health problems: a systematic and meta-analytic review. J Consult Clin Psychol. 2020;88(1):1-13. doi:10.1037/ccp0000459 31697093

