# Supplementary material for: Layperson-Delivered Telephone-Based Behavioral Activation Among Low-Income Older Adults During the COVID-19 Pandemic: The HEAL-HOA Randomized Clinical Trial
Source: JAMA Netw Open. 2024 Jun 18;7(6):e2416767. doi: 10.1001/jamanetworkopen.2024.16767 (PMC11185980; doi:10.1001/jamanetworkopen.2024.16767)
Supplement: Supplement 2. — eTable 1. Session Content of Tele-BA Intervention eTable 2. Session Content of Tele-MF Intervention eTable 3. Baseline Demographic and Clinical Characteristics Between Non-Completers and Completers (Per Protocol) eTable 4. Mixed-Effects Analysis on Primary Outcomes (Per Protocol) eTable 5. Mixed-Effects Analysis on Secondary Outcomes (Per Protocol) eFigure. Outcome Scores by Intervention Groups Across Assessment (Marginal Means) [file jamanetwopen-e2416767-s002.pdf]

Supplementary Online Content

Kwok JYY, Jiang D, Yeung DYL, et al. Layperson-delivered telephone-based behavioral activation among low-income older adults during the covid-19 pandemic: the HEAL-HOA randomized clinical trial. *JAMA Netw Open*. 2024;7(6):e2416767. doi:10.1001/jamanetworkopen.2024.16767

eTable 1. Session Content of Tele-BA Intervention

eTable 2. Session Content of Tele-MF Intervention

eTable 3. Baseline Demographic and Clinical Characteristics Between Non-Completers and Completers (Per Protocol)

eTable 4. Mixed-Effects Analysis on Primary Outcomes (Per Protocol)

eTable 5. Mixed-Effects Analysis on Secondary Outcomes (Per Protocol)

eFigure. Outcome Scores by Intervention Groups Across Assessment (Marginal Means)

This supplementary material has been provided by the authors to give readers additional information about their work.

© 2024 Kwok JYY et al. *JAMA Network Open*.

eTable 1. Session Content of Tele-BA Intervention

Session	Topic
1	Introduction to behavioral activation and record daily activity
2	Behavior monitoring, and identify important and meaningful life domains, values, and activities
3	Guide participants to understand what is important to them, and select and plan rewarding activities
4	Identify and overcome obstacles and seek for support for difficult activities
5	Guide participants to think about how to maintain gains after this intervention program
6	Reinforce behavior monitoring and activity planning in the coming week
7	Continue the discussion on problem solving and how to maintain gains after BA
8	Review BA learning goals and celebrate successes

eTable 2. Session Content of Tele-MF Intervention

Session	Topic
1	Introduction to mindfulness
2	Exercise on body focus techniques with closed and open eyes
3	Exercise on body focus techniques when listening to others talk
4	Exercise on body and mind focus techniques using verbal and mental labels of feel
5	Exercise on body focus techniques while maintaining equanimity by keeping their body relaxed
6	Exercise on distinguishing different types of sensations, levels of intensity, and spatial patterns and to detect subtle body events
7	Exercise on thoughts and alternative viewpoints and maintaining a positive attitude toward stress
8	Practice review, body scan, exercise on looking forward and preparing for the future

© 2024 Kwok JYY et al. *JAMA Network Open*.

eTable 3. Baseline Demographic and Clinical Characteristics Between Non-Completers and Completers (Per Protocol)

Characteristic	Participants, No (%)		*p-value*
	Non-completers[l] (n=134)	Completers[m] (n=573)	
Age (years), mean (SD)	75.60 (8.10)	75.69 (7.43)	0.90
Sex			0.22
Male	47 (35.1)	170 (29.7)	
Female	87 (64.9)	403 (70.3)	
Marital status			0.92
Married	10 (7.5)	43 (7.5)	
Single (never married)	18 (13.4)	79 (13.8)	
Widowed	61 (45.5)	276 (48.2)	
Divorced	45 (33.6)	175 (30.5)	
Level of education			0.71
None	28 (20.9)	145 (25.3)	
Primary or below	57 (42.5)	223 (38.9)	
Secondary	44 (32.8)	180 (31.4)	
Tertiary	5 (3.7)	25 (4.4)	
No of chronic diseases[a]			0.10
0	9 (6.7)	109 (19.0)	
1	26 (19.4)	111 (19.4)	
2	30 (22.4)	118 (20.6)	
≥ 3	69 (51.5)	235 (41.0)	
UCLA-L, mean (SD)[b]	50.9 (7.6)	51.0 (7.1)	0.82
DJGL, mean (SD)[c]	4.3 (1.3)	4.4 (1.5)	0.21
PHQ-9, mean (SD)[d]	1.8 (3.1)	2.5 (3.8)	0.05
HADS, mean (SD)[e]	1.9 (3.5)	2.2 (3.6)	0.38
PSS, mean (SD)[f]	9.3 (10.7)	14.5 (12.1)	<0.001
SWLS, mean (SD)[g]	19.4 (3.3)	19.1 (3.6)	0.52
PWB, mean (SD)[h]	56.6 (9)	56.3 (7.8)	0.62
SCI, mean (SD)[i]	23.1 (7.5)	21.9 (8.3)	0.14
MSPSS, mean (SD)[j]	38.1 (15.8)	39.8 (15.1)	0.24
LSNS, mean (SD)[k]	7.3 (5.6)	8.4 (6.1)	.04

Abbreviations: De Jong Gierveld Loneliness Scale; HADS-A, Hospital Anxiety and Depression Scale-Anxiety subscale; LSNS-6, Lubben Social Network Scale; MSPSS, Multidimensional Scale of Perceived Social Support; PHQ-9, 9-item Patient Health Questionnaire; PSS, Perceived Stress Scale; PWB, Psychological Well-being scale; SCI, Sleep Condition Indicator; SWLS, Satisfaction with Life Scale; UCLA-L, UCLA Loneliness scale.

[a]Included chronic non-specific lung disease, cardiac disease, peripheral disease, stroke, diabetes, arthritis, and cancer.

[b]UCLA-L scale ranges from 20 to 80, with higher scores indicating greater loneliness.

[c]DJGL scale ranges from 0 to 6, with higher scores indicating greater loneliness.

[d]PHQ-9 scale, a measure of severity of depressive symptoms, ranges from 0 to 27, with higher scores indicating more severe depressive symptoms.

[e]HADS-A subscale, a measure of severity anxiety symptoms, ranges from 0 to 21, higher scores indicate higher levels of anxiety.

[f]PSS, a measure of perceived stress, ranges from 0 to 56, with higher scores indicating higher levels of stress.

[g]SWLS, a measure of satisfaction with life, ranges from 5 to 35, with higher scores indicating higher levels of life satisfaction.

[h]PWB, a measure of different aspects of wellbeing and happiness, ranges from 16 to 96, with higher scores indicating better psychological wellbeing.

[i]SCI, a measure of sleeping condition and evaluating insomnia disorder, ranges from 0 to 32, with higher scores indicating better sleeping quality.

[j]MSPSS, a measure of perceived social support, ranges from 12 to 84, with higher scores indicating greater perceived social support.

[k]LSNS-6, a measure of social network, ranges from 0 to 30, with higher scores indicating more social engagement.

[l]Non-completers are defined as participants who attended only time 1 (1 month assessment), only time 2 (3 months assessment), or neither time 1 (1 month assessment) nor time 2 (3 months assessment).

[m]Completers are defined as participants who attended both the time 1 (1 month) and time 2 (3 months) follow-up assessments.

© 2024 Kwok JYY et al. *JAMA Network Open*.

eTable 4. Mixed-Effects Analysis on Primary Outcomes (Per Protocol)

Measures	Estimates across all follow ups, mean (95% CI)[a-d]						Tele-BA vs Tele-BF			Tele-MF vs Tele-BF			Overall between-group difference, *p-value*
	Tele-BA	*p-value*[1, 2]	Tele-MF	*p-value*[1, 2]	Tele-BF	*p-value*[1, 2]	Mean difference (Tele-BA Tele-BF) (95% CI)	Cohen's d	Mean difference *p-value*	Mean difference (Tele-MF - Tele-BF) (95% CI)	Cohen's d	Mean difference *p-value*	
UCLA-L[e]													
T0	50.37 (49.37 to 51.37)	NA	51.31 (50.41 to 52.20)	NA	51.43 (50.48 to 52.39)	NA	NA	NA	NA	NA	NA	NA	NA
T1	49.07 (48.09 to 50.05)	<.001	49.37 (48.41 to 50.33)	<.001	50.51 (49.66 to 51.36)	<.001	-1.44 (-3.03 to 0.14)	0.22	0.09	-1.14 (-2.71 to 0.43)	0.26	0.24	0.009
T2	48.38 (47.33 to 49.43)	<.001	49.03 (48.04 to 50.01)	<.001	50.79 (49.88 to 51.7)	0.12	-2.41 (-4.11 to -0.71)	0.42	0.002	-1.77 (-3.41 to -0.13)	0.32	0.03	<.001
DJGL[f]													
T0	4.49 (4.28 to 4.69)	NA	4.67 (4.46 to 4.88)	NA	4.21 (4.04 to 4.39)	NA	NA	NA	NA	NA	NA	NA	NA
T1	4.20 (4.02 to 4.37)	<.001	4.39 (4.22 to 4.56)	<.001	3.99 (3.84 to 4.14)	<.001	0.21 (-0.08 to 0.49)	0.09	0.25	0.40 (0.12 to 0.68)	0.23	0.002	0.66
T2	4.08 (3.89 to 4.26)	<.001	4.31 (4.14 to 4.49)	<.001	4.09 (3.93 to 4.25)	0.01	-0.01 (-0.31 to 0.29)	0.30	>.99	0.22 (-0.07 to 0.51)	0.09	0.19	<.001

Abbreviations: De Jong Gierveld Loneliness Scale; NA, Not Applicable; UCLA-L, UCLA Loneliness scale.
[a]631 participants completed at least 75% of sessions and completed time 1 (1 month) follow-up assessments; and 573 participants completed at least 75% of sessions and completed time 2 (3 months) follow-up assessments.
[b]All p-values reported in the analysis are non-adjusted. In this study, a p-value threshold of less than .0125 will be considered statistically significant.
[c]Paired t-test between scores at time 0 (baseline) and time 1 (1 month assessment) for each intervention group separately.
[d]Participants completed at least 75% of sessions and completed time 2 (3 months) follow-up assessments (n=573)
[e]UCLA-L scale ranges from 20 to 80, with higher scores indicating greater loneliness.
[f]DJGL scale ranges from 0 to 6, with higher scores indicating greater loneliness.

© 2024 Kwok JYY et al. *JAMA Network Open*.

eTable 5. Mixed-Effects Analysis on Secondary Outcomes (Per Protocol)

Measures	Estimates across all follow ups, mean (95% CI)[a-d]						Tele-BA vs Tele-BF			Tele-MF vs Tele-BF			Overall between-group difference, *p-value*
	Tele-BA	*p-value*[1, 2]	Tele-MF	*p-value*[1, 2]	Tele-BF	*p-value*[1, 2]	Mean difference (Tele-BA - Tele-BF) (95% CI)	Cohen's d	Mean difference *p-value*	Mean difference (Tele-MF - Tele-BF) (95% CI)	Cohen's d	Mean difference *p-value*	
PHQ9[e]													
T0	1.99 (1.51 to 2.48)	NA	2.35 (1.83 to 2.88)	NA	2.72 (2.22 to 3.22)	NA	NA	NA	NA	NA	NA	NA	NA
T1	2.26 (1.79 to 2.74)	0.03	2.61 (2.14 to 3.07)	0.03	2.95 (2.53 to 3.36)	0.05	-0.69 (-1.46 to 0.08)	0.18	0.10	-0.34 (-1.10 to 0.42)	0.081	0.85	0.98
T2	2.27 (1.81 to 2.73)	0.38	2.56 (2.13 to 2.99)	0.78	3.05 (2.65 to 3.44)	0.55	-0.77 (-1.51 to -0.03)	0.21	0.04	-0.49 (-1.20 to 0.23)	0.179	0.31	0.66
HADS-Anxiety[f]													
T0	1.83 (1.33 to 2.33)	NA	2.34 (1.84 to 2.84)	NA	2.31 (1.86 to 2.76)	NA	NA	NA	NA	NA	NA	NA	NA
T1	1.80 (1.38 to 2.22)	0.83	2.22 (1.81 to 2.63)	0.36	2.42 (2.05 to 2.78)	0.38	-0.61 (-1.29 to 0.07)	0.23	0.10	-0.19 (-0.87 to 0.48)	0.116	>.99	0.43
T2	1.85 (1.44 to 2.25)	0.80	2.26 (1.88 to 2.64)	0.68	2.50 (2.15 to 2.85)	0.67	-0.65 (-1.31 to 0.01)	0.19	0.05	-0.24 (-0.88 to 0.40)	0.053	>.99	0.80
PSS[g]													
T0	14.49 (12.76 to 16.21)	NA	17.36 (15.67 to 19.05)	NA	10.70 (9.32 to 12.08)	NA	NA	NA	NA	NA	NA	NA	NA
T1	13.59 (12.06 to 15.13)	<0.001	16.56 (15.05 to 18.06)	<0.001	10.56 (9.22 to 11.90)	0.55	3.03 (0.54 to 5.52)	0.21	0.01	5.99 (3.53 to 8.45)	0.481	<0.001	0.03
T2	14.24 (12.68 to 15.81)	0.02	16.64 (15.18 to 18.11)	0.006	11.01 (9.66 to 12.36)	0.63	3.24 (0.71 to 5.76)	0.24	0.007	5.64 (3.20 to 8.07)	0.459	<0.001	0.03
SWLS[h]													
T0	19.40 (18.91 to 19.89)	NA	18.67 (18.14 to 19.2)	NA	19.30 (18.87 to 19.72)	NA	NA	NA	NA	NA	NA	NA	NA
T1	19.82 (19.37 to 20.28)	<0.001	19.27 (18.82 to 19.72)	<0.001	19.52 (19.12 to 19.92)	0.09	0.30 (-0.44 to 1.05)	0.14	0.98	-0.25 (-0.98 to 0.48)	0.032	>.99	0.10
T2	20.23 (19.74 to 20.72)	<0.001	19.66 (19.20 to 20.11)	<0.001	19.43 (19.01 to 19.85)	0.28	0.80 (0.01 to 1.59)	0.41	0.05	0.23 (-0.53 to 0.99)	0.268	>.99	<0.001
PWB[i]													
T0	57.22 (56.06 to 58.38)	NA	55.37 (54.41 to 56.33)	NA	56.29 (55.23 to 57.35)	NA	NA	NA	NA	NA	NA	NA	NA
T1	58.80 (57.72 to 59.88)	<0.001	57.11 (56.05 to 58.17)	<0.001	57.51 (56.57 to 58.46)	<0.001	1.29 (-0.46 to 3.04)	0.18	0.23	-0.40 (-2.13 to 1.33)	0.013	>.99	0.34
T2	59.50 (58.37 to 60.62)	<0.001	57.49 (56.43 to 58.54)	<0.001	57.18 (56.21 to 58.15)	0.20	2.32 (0.50 to 4.13)	0.41	0.007	0.31 (-1.45 to 2.06)	0.207	>.99	<0.001
SCI[j]													
T0	22.99 (21.84 to 24.14)	NA	22.88 (21.82 to 23.95)	NA	20.54 (19.46 to 21.62)	NA	NA	NA	NA	NA	NA	NA	NA
T1	22.92 (21.86 to 23.97)	0.75	22.83 (21.80 to 23.86)	0.84	20.15 (19.23 to 21.07)	0.09	2.76 (1.06 to 4.47)	0.38	<0.001	2.68 (1.00 to 4.37)	0.365	<0.001	0.50
T2	22.76 (21.72 to 23.80)	0.69	22.65 (21.67 to 23.62)	0.64	20.21 (19.31 to 21.11)	0.97	2.55 (0.87 to 4.23)	0.26	0.001	2.44 (0.82 to 4.06)	0.242	0.001	0.91
MSPSS[k]													
T0	41.49 (39.33, 43.65)	NA	39.24 (37.38, 41.11)	NA	38.55 (36.5, 40.61)	NA	NA	NA	NA	NA	NA	NA	NA
T1	42.60 (40.48, 44.73)	0.006	40.72 (38.64, 42.81)	<0.001	39.93 (38.07, 41.78)	<0.001	2.68 (-0.77, 6.13)	0.14	0.19	0.80 (-2.61, 4.21)	0.05	>.99	0.82
T2	44.01 (41.84, 46.19)	0.003	41.93 (39.90, 43.97)	<0.001	39.76 (37.88, 41.64)	0.13	4.25 (0.75, 7.76)	0.37	0.01	2.17 (-1.21, 5.56)	0.27	0.37	0.008
LSNS-6[l]													
T0	8.52 (7.69, 9.35)	NA	7.89 (7.04, 8.74)	NA	8.51 (7.74, 9.29)	NA	NA	NA	NA	NA	NA	NA	NA
T1	8.77 (7.96, 9.58)	0.14	8.23 (7.44, 9.02)	0.07	8.76 (8.05, 9.46)	0.09	0.01 (-1.3, 1.32)	0.00	>.99	-0.53 (-1.82, 0.77)	0.07	0.99	0.89
T2	9.33 (8.53, 10.13)	0.005	8.78 (8.02, 9.53)	<0.001	8.65 (7.95, 9.34)	0.69	0.68 (-0.62, 1.98)	0.25	0.63	0.13 (-1.13, 1.39)	0.22	>.99	0.001

Abbreviations: HADS-A, Hospital Anxiety and Depression Scale-Anxiety subscale; LSNS-6, Lubben Social Network Scale; MSPSS, Multidimensional Scale of Perceived Social Support; NA, Not Applicable; PHQ-9, 9-item Patient Health Questionnaire; PSS, Perceived Stress Scale; PWB, Psychological Well-being scale; SCI, Sleep Condition Indicator; SWLS, Satisfaction with Life Scale.

[a]631 participants completed at least 75% of sessions and completed time 1 (1 month) follow-up assessments; and 573 participants completed at least 75% of sessions and completed time 2 (3 months) follow-up assessments.

© 2024 Kwok JYY et al. *JAMA Network Open*.

[b]All p-values reported in the analysis are non-adjusted. In this study, a p-value threshold of less than .0125 will be considered statistically significant.
[c]Paired t-test between scores at time 0 (baseline) and time 1 (1 month assessment) for each intervention group separately.
[d]Participants completed at least 75% of sessions and completed time 2 (3 months) assessments (n=573)
[e]PHQ-9 scale, a measure of severity of depressive symptoms, ranges from 0 to 27, with higher scores indicating more severe depressive symptoms.
[f]HADS-A subscale, a measure of severity anxiety symptoms, ranges from 0 to 21, higher scores indicate higher levels of anxiety.
[g]PSS, a measure of perceived stress, ranges from 0 to 56, with higher scores indicating higher levels of stress.
[h]SWLS, a measure of satisfaction with life, ranges from 5 to 35, with higher scores indicating higher levels of life satisfaction.
[i]PWB, a measure of different aspects of wellbeing and happiness, ranges from 16 to 96, with higher scores indicating better psychological wellbeing.
[j]SCI, a measure of sleeping condition and evaluating insomnia disorder, ranges from 0 to 32, with higher scores indicating better sleeping quality.
[k]MSPSS, a measure of perceived social support, ranges from 12 to 84, with higher scores indicating greater perceived social support.
[l]LSNS-6, a measure of social network, ranges from 0 to 30, with higher scores indicating more social engagement.

© 2024 Kwok JYY et al. *JAMA Network Open*.

eFigure. Outcome Scores by Intervention Groups Across Assessment (Marginal Means)

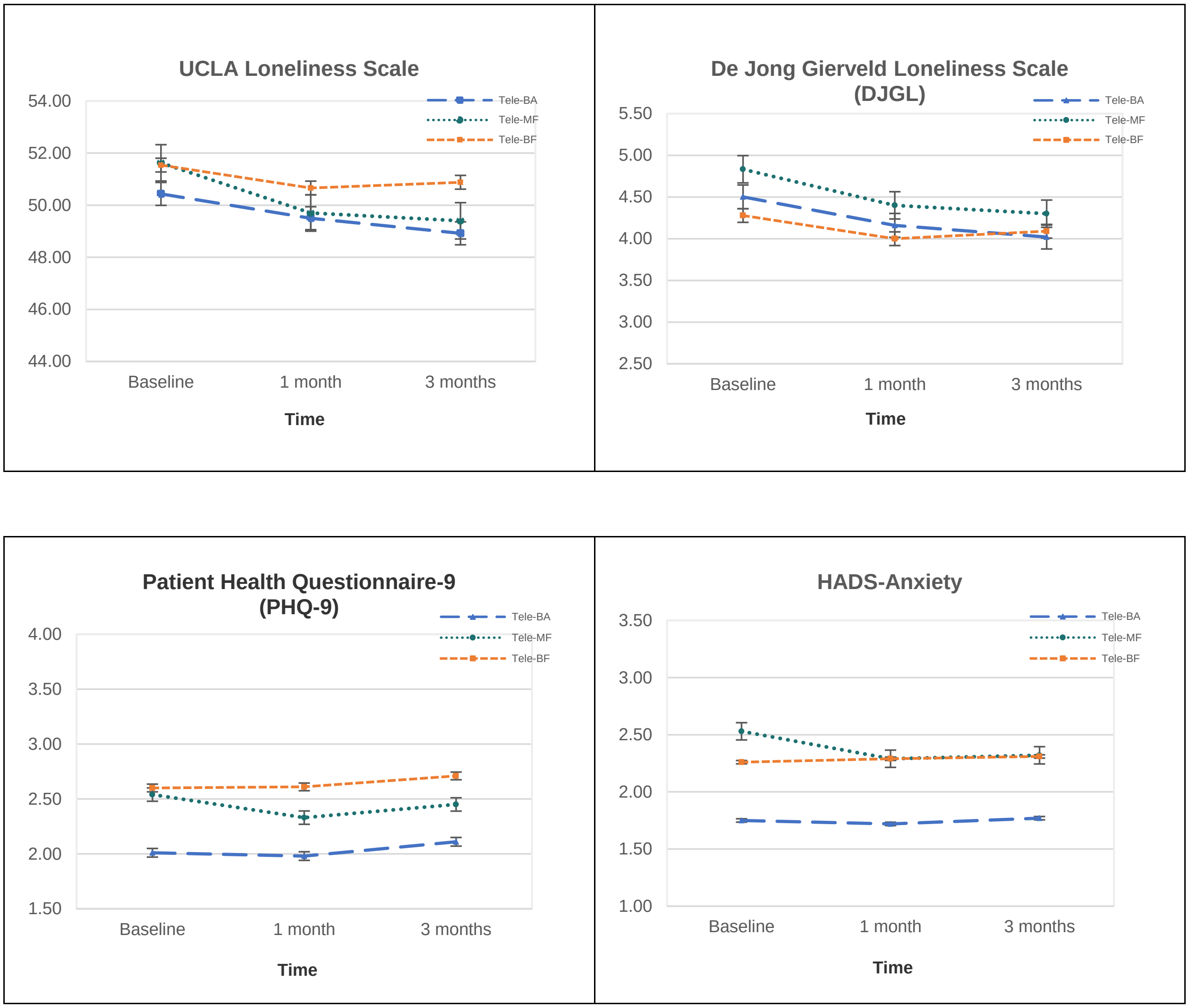

© 2024 Kwok JYY et al. *JAMA Network Open*.

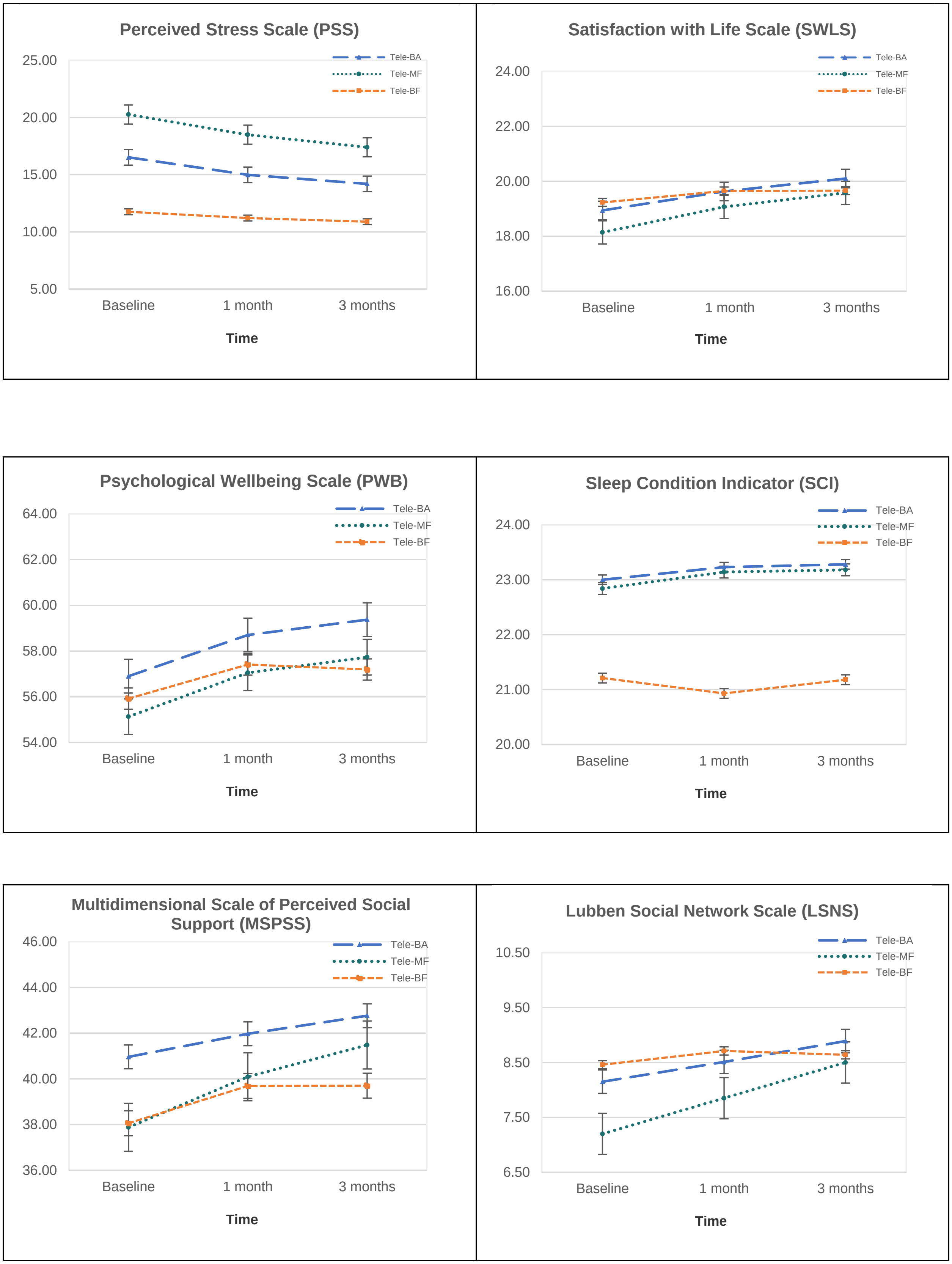

© 2024 Kwok JYY et al. *JAMA Network Open*.
