# Supplementary material for: Layperson-Delivered Telephone-Based Behavioral Activation Among Low-Income Older Adults During the COVID-19 Pandemic: The HEAL-HOA Randomized Clinical Trial
Source: JAMA Netw Open. 2024 Jun 18;7(6):e2416767. doi: 10.1001/jamanetworkopen.2024.16767 (PMC11185980; doi:10.1001/jamanetworkopen.2024.16767)
Supplement: Supplement 3. — Data Sharing Statement [file jamanetwopen-e2416767-s003.pdf]

Data Sharing Statement

Kwok. Layperson-Delivered Telephone-Based Behavioral Activation Among Low-Income Older Adults During the COVID-19 Pandemic. *JAMA Netw Open*. Published June 18, 2024. doi:10.1001/jamanetworkopen.2024.16767

Data
Data available: No

Additional Information
Explanation for why data not available: The data supporting the findings of this study are not publicly available due to the presence of personal information that could potentially compromise the privacy of the research participants but are available from KL Chou upon reasonable request.
